# Transcriptional Modulation of Heat-Shock Protein Gene Expression

**DOI:** 10.1155/2011/238601

**Published:** 2010-09-15

**Authors:** Anastasis Stephanou, David S. Latchman

**Affiliations:** Medical Molecular Biology Unit, Institute of Child Health, University College London, 30 Guilford Street, London WC1N 1EH, UK

## Abstract

Heat-shock proteins (Hsps) are molecular chaperones that are ubiquitously expressed but are also induced in cells exposed to stressful stimuli. Hsps have been implicated in the induction and propagation of several diseases. This paper focuses on regulatory factors that control the transcription of the genes encoding Hsps. We also highlight how distinct transcription factors are able to interact and modulate Hsps in different pathological states. Thus, a better understanding of the complex signaling pathways regulating Hsp expression may lead to novel therapeutic targets.

## 1. Introduction

The heat-shock proteins (Hsps) are a group of highly conserved proteins with major physiological roles in protein homeostasis [1, 2]. In most cell types even prior to stress Hsps constitute 1%-2% of total protein, suggesting an important role for these proteins in the biology and physiology of the unstressed cell. These particularly concern regulating the folding and unfolding of other proteins. The proteins are named, however, because they were first identified on the basis of their increased synthesis following exposure to elevated temperatures [3]. Subsequently it has been clearly shown that they can be induced following a variety of stressful stimuli. Some Hsps, such as Hsp90 (each Hsp is named according to its mass in kilodaltons) are detectable at significant levels in unstressed cells, increasing in abundance following a suitable stimulus, whilst others such as Hsp70 exist in both constitutively expressed and inducible forms that is activated by stressful stimuli [4, 5].

The dual role of Hsps in both normal and stressed cells, evidently requires the existence of complex regulatory processes which ensure that the correct expression pattern is produced. Indeed, such processes must be operative at the very earliest stages of embryonic development since the genes encoding Hsp70 and Hsp90 have been shown to be amongst the first embryonic genes which are transcribed [6, 7]. 

The induction of Hsps in response to various stresses is dependent on the activation of specific members of a family of transcription factors, the heat-shock factors (HSFs) which bind to the heat-shock element (HSE) in the promoters of the genes encoding Hsps [8]. Four HSFs (HSF1 to −4) have been cloned from a number of organisms and their roles have now been characterised. Only HSF1 and HSF3 have been shown to be involved in regulating Hsps in response to thermal stress whereas HSF2 and HSF4 are involved in Hsp regulation in unstressed cells and their levels are regulated in response to a wide variety of biological processes such as immune activation and cellular differentiation [8]. In general, however, the stimuli which induce such alterations in Hsp gene expression under nonstress conditions are poorly characterized and the mechanisms by which they act are unclear. In this paper, we discuss recent studies indicating that Hsps are not only regulated by HSFs alone, but also by transcription factors which are able to interact or cooperate with HSF1 and modulate the transcriptional regulation of Hsps in response to nonstressful stimuli. More recently, as will be explained later, it has also been reported that HSF2, like HSF1 can also play a role as a stress-inducible factor in promoting the induction of Hsps under certain conditions.

## 2. Transcriptional Regulation of Hsps by the HSF Family

### 2.1. HSF1

As mentioned previously, HSF1 has been identified as the HSF that mediates stress-induced Hsp gene expression in response to environmental stressors. Such stresses cause HSF1 oligomerization and nuclear translocalization, followed by enhanced DNA binding on the Hsp gene promoters. Recent studies have shown that HSF1 is negatively regulated by Hsp70 and Hsp90, therefore suggesting a negative-feedback loop for the regulation of Hsp70 and Hsp90 genes following a heat-shock response [8–10]. HSF1 is known to undergo posttranslational modification by various processes including phosphorylation, acetylation, and sumoylation [8]. Both phosphorylation and sumoylation are involved in regulating the transactivation capacity of HSF1 [8]. More recent, whereas p300 has been shown to acetylate HSF1, deacetylation by the NAD+-dependent sirtuin (SIRT1) is involved in the attenuation phase of the heat-shock response by preventing HSF1 acetylation and DNA binding [8].

The kinases responsible for phosphorylating HSF1 on several serine sites include glycogen synthase kinase 3*β* *(*GSK*β*) and c-jun N-terminal kinase (JNK) [11, 12]. The cytokine interleukin 6 (IL-6) has been shown to derepress HSF1 by reducing the activity of GSK*β* [13]. However, a positive role of HSF1 phosphorylation in the stress-induced activation of Hsp gene expression is also known to occur. The exact mechanism of this effect has not been fully elucidated, although the protein kinase CK2 seems to be involved in enhancing transcriptional activity and the DNA binding of HSF1 by phosphorylating the threonine 142 residue [14]. It is suggested that activation may also involve dephosphorylation of HSF1 [11].

The key role of HSF1 has been supported by the findings that cells lacking this crucial factor exhibited defects in Hsp induction following exposure to heat shock [15]. Moreover, cells lacking HSF1 were susceptible to apoptotic cell death following exposure to heat stress [15]. In addition, mice lacking HSF1 also had elevated levels of tumour necrosis factor *α* (TNF-*α*), which resulted in increased mortality after endotoxin and inflammatory challenge [15]. Interestingly, HSF1 has been shown to also modulate other genes such as interleukin-1*β* and c-fos [16, 17], suggesting a role for HSF1 in regulating stress responsive genes other than those encoding Hsps. 

More recently, it has been reported that HSF1 also functions in the circadian clock as a circadian transcription factor. The circadian clock enables an organism to adapt to conditions by presetting the area in the brain that controls behavioural changes. Circadian transcription factors are known to be regulated in a timely and rhythmic fashion Thus, using a novel technique of differential display of DNA-binding proteins (DDDPs), HSF1 was shown to be highly rhythmic in its transcriptional activity. Moreover, HSF1 enhanced the expression of Hsps at the onset of the dark phase, when the animals start to be behaviourally active. Furthermore, Hsf1-deficient mice have a longer free-running period and therefore more active than wild-type littermates, suggesting a combined role for HSF1 in the mammalian timekeeping and cytoprotection systems [18].

### 2.2. HSF2

As mentioned earlier, Hsp gene expression is crucial not only for the survival of cells exposed to extracellular stress stimuli, but also during normal cellular processes such as embryonic development and cellular differentiation. HSF2 has now been described as the factor involved in regulating Hsps under nonstressful conditions. For example, it was previously reported that Hsp70 expression is activated when K562 cells are induced by hemin and this process requires activation of HSF2 [19]. HSF2 exists as two isoforms, HSF2*α* and HSF2*β*, due to alternative splicing, where the HSF2*α* isoform is predominantly expressed in adult tissue, while the HSF2*β* isoform is predominantly expressed in embryonic tissue [20]. HSF2 DNA binding activity is high during early embryogenesis in tissues such as the heart, central nervous system, and testis [20]. The importance of HSF2 in development was recently reported and Hsf2-null mice display gametogenesis defects and brain abnormalities characterized by enlarged ventricles [21].

During mitosis, the genome is well known to be compacted in order for chromosomes to be segregated during cytokinesis. However, some gene promoters such as the inducible Hsp70i (heat stress-induced upregulation) remain uncompacted. The factors that control and prevent this process of compaction or bookmarking have been recently characterized. For example, Hsp70i bookmarking is now known to be mediated by HSF2, which binds this promoter in mitotic cells, recruits protein phosphatase 2A, and interacts with the CAP-G subunit of the condensin enzyme to promote efficient dephosphorylation and inactivation of condensin complexes in the vicinity, thereby preventing compaction at this site [22]. Blocking HSF2-mediated bookmarking by HSF2 RNA interference decreases hsp70i induction and survival of stressed cells in the G1 phase, which demonstrates the biological importance of gene bookmarking. HSF2 has also been shown to be bound to the HSE promoter elements of other heat-shock genes, including Hsp90 and Hsp27, as well as the proto-oncogene c-fos [23]. These data suggest that HSF2 is important for constitutive as well as stress-inducible expression of HSE-containing genes.

It is also known that HSF2 can form heterotrimers with HSF1. Following certain stress, HSF1 is activated and HSF1-HSF2 heterotrimers are formed. Heat-shock stress diminishes the levels of HSF2 and restricts heterotrimerization by limiting the availability of HSF2. It has been suggested that HSF1-HSF2 heterotrimerization provides a switch that integrates the transcriptional activation in response to specific stimuli during developmental processes; for review see [8]. 

### 2.3. HSF3

HSF3 was originally identified in avian cells and no reports have yet described HSF3 in other organisms. Like HSF1, HSF3 is also heat-stress responsive [24]. However, the threshold temperature required to activate HSF3 and HSF1 are different in that HSF1 is activated by less severe heat shock than HSF3 [24]. Previously, HSF3 was reported to bind to c-Myb, a transcription factor involved in cellular proliferation and required for the G1/S transition of the cell cycle, which also paralleled the expression of Hsp70 [25]. These studies suggest that HSF3/cMyb interaction may be involved in cell cycle-dependent expression of Hsps. Furthermore, more recently, it has been shown that HSF3/c-Myb association is disrupted by direct binding of the p53 tumour suppressor transcription factor to HSF3, resulting in inhibition of Hsp70 expression [26].

### 2.4. HSF4

In contrast to HSF1 and HSF2 proteins, which are expressed in most tissues, the level of HSF4 protein is very low in many mammalian tissues except in lung and brain [27]. There are at least two isoforms, HSF4a and HSF4b, which are derived by alternative RNA-splicing events. HSF4b is able to activate transcription whereas HSF4a does not and this differential effect has not yet been characterized [27, 28]. Interestingly, mutations of HSF4 have been associated with dominant inherited cataracts in human [29]. More recently, HSF4 has been revealed to have a role in regulating lens-specific gamma-crystalline genes during lens development [30].

## 3. The Role of Non-HSF Transcription Factors in Modulating Hsp Gene Expression by the STAT and NF-IL6 Pathways

The phenotype of mice lacking HSF1 is normal in the absence of stress and expression of Hsp70 and Hsp90 in cells lacking HSF1 is similar to wild-type cells, although they exhibit a defect in the heat-shock response following heat stress [13]. These studies suggest that other HSFs may compensate for the lack of HSF1 and/or that other transcription factors may also be responsible for expression of Hsps under normal growth conditions. Recent studies from our laboratory have identified a separate group of transcription factors that are activated by distinct cytokines and are able to modulate Hsp70 and Hsp90 gene expression. These factors include STAT1, STAT3, and NF-IL6 and their functional roles are described below.

The STATs are a family of cytoplasmic transcription factors that mediate intracellular signalling initiated at cytokine cell surface receptors and transmitted to the nucleus. STATs are activated by phosphorylation on conserved tyrosine and serine residues on their C-terminal domains by the Janus kinases (JAKs) and MAP kinase families, respectively. This allows the STATs to dimerise and translocate to the nucleus and thereby regulate gene expression [31]. Interferon-*γ* is a potent activator of STAT1, whilst the interleukin-6 (IL-6) family members including IL-6, leukaemia inhibitory factor (LIF), and CT-1 primarily activate STAT3 [31].

Our laboratory has previously shown that STAT1 and STAT3 have opposing actions on apoptotic cell death in various cell types [32]. We reported that overexpression of STAT1 is able to enhance apoptotic cell death in cardiac myocytes exposed to ischaemia reperfusion (I/R) injury whereas overexpression of STAT3 with STAT1 is able to reduce the levels of STAT1-induced cell death following I/R by modulating the expression of pro- and antiapoptotic genes [33]. Furthermore, these effects on apoptosis require serine-^727^ but not tyrosine-^701^ phosphorylation on the C-terminal transactivation domain of STAT1 [34, 35]. 

Moreover, we have shown that STAT1 is able to modulate the activity of p53 and its effects on apoptosis [36]. These effects involve STAT1/p53 protein-protein interaction with STAT1 acting as a coactivator for p53 [36]. We have also demonstrated that STAT1 is also able to interact with another p53 family member p73 [37]. However, in contrast to STAT1-p53 interaction which enhances p53 transcriptional activity, the STAT1-p73 interaction was shown to reduce p73 functional activity on similar p53-responsive genes [37]. Thus, STAT1 is able to have differential effects on p53/p73 transcriptional activity. 

A link between p53 activity and the HSF1-heat-shock response pathway has recently been documented by the finding that HSF1 interacts with stress-responsive activator of p300 (Strap) transcription cofactor, a key factor controlling the DNA damage response through its ability to regulate p53 activity [38]. Moreover, Strap augments HSF1 binding and chromatin acetylation in Hsp genes, most probably through the p300 histone acetyltransferase activity of p300 itself. Furthermore, cells depleted of Strap do not survive under heat-shock conditions [38]. Overall, these data indicate that Strap is an essential cofactor that acts at the level of chromatin control to regulate heat-shock-responsive transcription. 

The cytokine IL-6 is known to stimulate two distinct signalling pathways, resulting in the activation of two different classes of cellular transcription factors [39]. Thus, initial studies showed that a variety of IL-6-inducible genes contained binding sites for a transcription factor named NF-IL6 (nuclear factor IL-6), which showed high homology with the rat-liver nuclear factor C/EBP (CCAAT-enhancer-binding protein), and is therefore also known as C/EBP*β* [40]. Subsequently, another member of the C/EBP family, known as NF-IL*β* or C/EBP*δ*, was identified and shown to form heterodimers with NF-IL6, resulting in a synergistic transcriptional effect [41]. After exposure of cells to IL-6, NF-IL6 is phosphorylated, resulting in its enhanced ability to stimulate transcription [41] whereas NF-IL6*β* is synthesized *de novo* [41]. As mentioned above the second pathway which is stimulated by IL-6 is the JAK/STAT3 signalling pathway. 

It is generally accepted that the NF-IL6/NF-IL6*β* and STAT3-signalling pathways allow IL-6 to activate two distinct sets of genes, each of which is responsive to one of these pathways. Thus, class 1 acute-phase proteins (such as *α*_1_-acid glycoprotein, haptoglobin, C-reactive protein, and serum amyloid) contain response elements for NF-IL6 and NF-IL6*β* and these factors have been shown to be involved in the activation of these genes following IL-6 treatment [32]. In agreement with this idea, these genes are stimulated by exposure of cells to IL-1 which also stimulates NF-IL6/NF-IL6*β* activity without affecting STAT3 [42]. In contrast, type 2 acute-phase genes such as fibrinogen, thiostatin, and *α*_2_ microglobulin are not inducible by IL-1 and lack binding sites for NF-IL6/NF-IL6*β*. Instead, these genes contain binding sites allowing binding of STAT3, which is responsible for activation of these genes in response to IL-6 [42].

## 4. Role of STAT1, STAT3, and NF-IL6 Factors in Modulating Hsps

We previously reported [43] that IL-6 can induce increased expression of the 90 kDa heat-shock protein (hsp90) in a variety of different cell types. The hsp90*β* gene promoter was shown to be responsive to IL-6 and could also be activated by NF-IL6 or NF-IL6*β*  [43] . Moreover, a short region of the promoter containing an NF-IL6-binding site was essential for activation of the promoter by both IL-6 and NF-IL6. This promoter region could confer responsiveness both to IL-6 and to overexpression of NF-IL6 on a heterologous promoter. These findings suggested that hsp90 was a member of the class of IL-6-responsive genes that were activated by NF-IL6/NF-IL6*β*. 

Interestingly, this short region of the promoter also contains binding sites for STAT3 and the hsp90 promoter can be activated also by this factor. Moreover, overexpression of NF-IL6 and STAT3 has a synergistic effect on the hsp90 promoter and both these signalling pathways appear to be required for activation of the hsp90 promoter by IL-6 [44]. Despite their synergistic action in IL-6 signalling, however, these two pathways have opposite effects on the heat-shock-mediated regulation of the hsp90 promoter. Thus STAT3 reduces the stimulatory effect of heat shock whereas NF-IL6 enhances it. When applied together, heat shock and IL-6 produce only weak activation of the hsp90 promoter compared with either stimulus alone, indicating that the inhibitory effect of STAT3 on HSF predominates under these conditions [44]. In contrast, IL-1, which activates only the NF-IL6 pathway, synergizes with heat shock to produce strong activation of hsp90 [44]. These results therefore open up a new aspect of hsp90-gene regulation which is additional to and interacts with the heat-shock-activated pathway. 

Previously, we had also examined whether STAT1 is able to modulate Hsp expression. We showed that IFN-*γ* treatment increases the levels of Hsp-70 and Hsp-90 and also enhances the activity of the Hsp-70 and Hsp-90*β* promoters with these effects being dependent on activation of the STAT1 transcription factor by IFN-*γ* [45]. These effects were not seen in a STAT1-deficient cell line, indicating that IFN-*γ* modulates Hsp induction via a STAT1-dependent pathway. The effect of IFN-*γ*/STAT1 was mediated via the same short region of the Hsp-70/Hsp-90 promoters, which also mediates the effects of NF-IL6 and STAT3 and can bind STAT1 [45]. 

This region also contains a binding site for the stress-activated transcription factor HSF1. We showed that STAT1 and HSF1 interact with one another via a protein-protein interaction and produce a strong activation of transcription [45]. This is in contrast to the previous finding that STAT3 and HSF1 antagonize one another and we showed that STAT3 and HSF1 do not interact directly. To our knowledge, this was the first report of HSF1 interacting directly via a protein-protein interaction with another transcription factor. Such protein-protein interactions and the binding of a number of different stress and cytokine-activated transcription factors to a short region of the Hsp-90 and Hsp-70 gene promoters are likely to play a very important role in Hsp gene activation by nonstressful stimuli and the integration of these responses with the stress response of these genes. Moreover, our findings that STAT1 can interact with p53 and that both these factors are able to modulate the effects of HSF1 on Hsp expression, suggests different interacting partners of HSF1 may affect HSF1-mediated transcriptional regulation.

## 5. Linking HSF1, STAT1, STAT3, and NF-IL6 Elevation to Pathological Diseases

A number of disease states have been shown to exhibit elevated levels of Hsps [46]. This includes patients with systemic lupus erythematosus (SLE) who have elevated levels of Hsp90. Interestingly, elevated levels of circulating IL-6 have also been reported in SLE [47], and the levels have been shown to be correlated with disease activity, being highest in patients with active disease. Moreover, spontaneous production of IgG by normal and SLE-derived B lymphocytes in culture can be enhanced by the addition of exogenous IL-6 and inhibited by antibody to IL-6 [48]. These findings therefore suggest that IL-6 might play a role in the pathogenesis of autoimmune diseases. Moreover, infusion of an antibody to IL-6 can relieve disease symptoms in lupus-prone NZB/NZW F1 mice [49]. Furthermore, elevated levels of Hsp90 in SLE correlated with levels of IL-6 and of autoantibodies to Hsp90 [50].

In order therefore to test directly the role of IL-6 in regulating Hsp90 expression *in vivo* we have used mice which have been artificially engineered to express elevated levels of IL-6 either by being made transgenic for extra copies of the IL-6 gene [51] or by inactivation of the gene encoding the transcription factor C/EBP*β* which also results in the elevation of IL-6 levels in these mice [52]. In these experiments, elevated levels of Hsp90 were observed in both the IL-6 transgenic and the C/EBP*β* knock-out mice [53]. Hence, the elevated IL-6 levels induced in these animals are indeed paralleled by increased levels of Hsp90 compared to normal control mice. In addition, it was also observed that in both IL-6 transgenic and C/EBP*β* knock-out animals, elevated hsp90 was associated with the specific production of autoantibodies to Hsp90. It is also of interest that inactivation of the IL-6 gene in the C/EBP*β* knock-out mice resulted in the suppression of Castleman-like disease normally observed in these animals and a reduction in the production of autoantibodies. 

These results support a model in which elevated levels of IL-6 in SLE patients induce increased levels of Hsp90 protein which in turn results in the production of autoantibodies to this protein. Additionally, IL-10 is also elevated in SLE and IL-10 was demonstrated to enhance Hsp90 gene expression [54]. Therefore, these studies strongly suggest that IL-6 and IL-10 are likely to play a critical role in the regulation of Hsp90 levels and autoantibody production in autoimmune disease states.

As described above, NF-IL6 performs diverse functions, participating in the regulation of genes that contribute to the known acute phase response, but also to glucose metabolism, and tissue differentiation, including adipogenesis and hematopoiesis [55–57]. Hsps and STAT3 have also been found at increased levels in many solid tumours and haematological malignancies [58, 59]. Their expression may in part account for the ability of malignant cells to maintain protein homoeostasis even in the hostile hypoxic microenvironment of the tumour. Thus, STAT3 seems to function as an antiapoptotic factor, especially in numerous malignancies, where STAT3 is often constitutively active/phosphorylated and STAT3 activation has been associated with advanced stages of metastatic cancers such as prostate cancer [60]. Furthermore, STAT3 behaves as an oncogene, and is able to transform normal fibroblast cells which can then form tumours in nude mice [61]. Thus, targeting STAT3 activation has been suggested to be an attractive anticancer therapy [60]. 

Likewise, Hsps allow tumour cell survival, growth, and metastasis, even in growth factor-deprived conditions, by allowing continued protein translation and cellular proliferation [62, 63]. Therefore, targeting of Hsps with chemical inhibitors may be beneficial in multiple oncogenic processes. [64]. Further evidence for a link between Hsps and cancer was reported from studies in the HSF1 knockout mice, which showed reduced development of tumours, and HSF1 deficiency rendered cultured cells highly refractory to transformation initiated by mutated RAS or by platelet-derived growth factor-B (PDGF-B) overexpression [65, 66]. Similarly, HSF1 depletion decreased viability of multiple human cancer cell lines, but had no effect on normal cells, suggesting that HSF1 provides critical relief to the cellular stresses experienced by cancer cells [67]. It is therefore plausible that the STAT3-Hsp interactions may be one such survival pathway that allows tumour cell survival, growth and metastasis in cancers.

## 6. Conclusion

**Figure 1:**
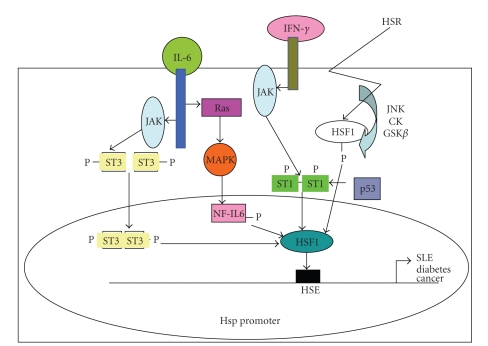
Signal transduction pathways activated by STAT1, STAT3, NF-IL6, p53, and the heat-shock response (HSR) via HSF1 binding to the heat-shock response element (HSE) and integrating to modulate Hsp transcription, which is known to be dysregulation in different pathological diseases.

This review paper demonstrates the modulation of Hsps by a group of transcription factors other than the traditional HSF family under normal nonstressful conditions and also in several disease states. The finding that the responses to these factors occur around the HSF DNA binding site in the Hsp gene promoters, suggests that HSF1 as well as other HSFs are able to interact or cooperate with STATs or NF-IL6 family members. Further studies to identify novel protein interacting partners for HSFs will also provide insight into the regulation of Hsps and other molecular chaperones. Unravelling the mechanistic basis of this cooperation will undoubtedly enhance our understanding of the interdependent relationship between distinct HSFs and their interaction with other factors in the complex regulatory processes which ensure that the correct Hsp expression pattern is produced under different physiological states (Figure 1).
